# RNAi-Related Dicer and Argonaute Proteins Play Critical Roles for Meiocyte Formation, Chromosome-Axes Lengths and Crossover Patterning in the Fungus *Sordaria macrospora*

**DOI:** 10.3389/fcell.2021.684108

**Published:** 2021-06-28

**Authors:** Chloe Girard, Karine Budin, Stéphanie Boisnard, Liangran Zhang, Robert Debuchy, Denise Zickler, Eric Espagne

**Affiliations:** ^1^Université Paris-Saclay, Commissariat à l’Énergie Atomiques et aux Énergies Alternatives (CEA), Centre National de la Recherche Scientifique (CNRS), Institute for Integrative Biology of the Cell (I2BC), Gif-sur-Yvette, France; ^2^Center for Reproductive Medicine, Cheeloo College of Medicine, Shandong University, Jinan, China

**Keywords:** RNAi, dicer, Argonaute (AGO), axis length, crossover patterning, meiosis

## Abstract

RNA interference (RNAi) is a cellular process involving small RNAs that target and regulate complementary RNA transcripts. This phenomenon has well-characterized roles in regulating gene and transposon expression. In addition, Dicer and Argonaute proteins, which are key players of RNAi, also have functions unrelated to gene repression. We show here that in the filamentous Ascomycete *Sordaria macrospora*, genes encoding the two Dicer (Dcl1 and Dcl2) and the two Argonaute (Sms2 and Qde2) proteins are dispensable for vegetative growth. However, we identified roles for all four proteins in the sexual cycle. Dcl1 and Sms2 are essential for timely and successful ascus/meiocyte formation. During meiosis *per se*, Dcl1, Dcl2, and Qde2 modulate, more or less severely, chromosome axis length and crossover numbers, patterning and interference. Additionally, Sms2 is necessary both for correct synaptonemal complex formation and loading of the pro-crossover E3 ligase-protein Hei10. Moreover, meiocyte formation, and thus meiotic induction, is completely blocked in the *dcl1 dcl2* and *dcl1 sms2* null double mutants. These results indicate complex roles of the RNAi machinery during major steps of the meiotic process with newly uncovered roles for chromosomes-axis length modulation and crossover patterning regulation.

## Introduction

Ribonucleic-acid silencing (or RNAi) was originally described as a post-transcriptional gene-silencing (PTGS) mechanism mediated by small RNAs. RNAi plays essential roles across eukaryotes in genome defense against transposons and viruses (e.g., Malone and Hannon, 2009) as well as during development and cellular differentiation (Alberti and Cochella, 2017; D’Ario et al., 2017). Proteins involved in RNAi are well conserved across kingdoms, and include three main components: an RNA-dependent RNA polymerase (RdRP) generating double stranded RNAs (dsRNAs), the type III RNase Dicer module dicing up precursor dsRNAs into small dsRNAs that are then loaded onto an Argonaute-PIWI protein to target complementary sequences [reviewed in Cerutti and Casas-Mollano (2006)]. The ancestral role of the RNAi machinery in the eukaryotic ancestor(s) may have been geared toward targeting RNA transcript degradation and locus-specific histone modifications to promote gene silencing (e.g., Gutbrod and Martienssen, 2020). However, findings in multiple organisms have now demonstrated the involvement of RNAi components in many other nuclear functions including centromere integrity, chromosome segregation and DNA repair [reviewed in Burger and Gullerova (2015) and Gutbrod and Martienssen (2020)].

Factors involved in RNAi are also known to regulate a wide variety of cellular processes during both the vegetative and the sexual cycles in the filamentous fungus *Neurospora crassa*, a close relative of *Sordaria macrospora* (Nowrousian et al., 2004). The first of those RNA silencing mechanisms, termed quelling, mediates post-transcriptional silencing of transgenes in mitotic cells (Romano and Macino, 1992). Quelling involves the QDE-1/2/3 proteins and QIP (QDE-2 interacting protein; Fulci and Macino, 2007). Mutants of the implicated factors, while affected in transgene silencing, show no developmental phenotypes, either vegetative or sexual. Subsequent work revealed, however, that sensitivity to DNA damaging agents was increased in these mutants, and that the Argonaute homolog QDE-2 was over-expressed after exposure to DNA damaging agents or in mutants of the DNA damage repair pathway (Lee et al., 2009). RNAi is also implicated in a second type of gene silencing called Meiotic Silencing by Unpaired DNA (MSUD). This process occurs exclusively during meiotic prophase. It scans homologous chromosomes for unpaired DNA sequences and silences both the unpaired and any other homologous copies present in the genome (Shiu et al., 2001). How unpaired copies are detected remains unknown, but this process requires at least nine proteins, including three RNAi proteins: the Dicer homolog Dcl-1, the Argonaute protein Sms-2 and the RNA-dependent RNA polymerase [reviewed in Hammond (2017)]. Dicer and Argonaute proteins are also involved during meiosis in other fungi, as well as in mammals and plants. In the fission yeast *Schizosaccharomyces pombe*, both *dcr1* and *ago1* mutants show chromosome mis-segregation during the second meiotic division (Hall et al., 2003). Mammalian Dicer- and Dgcr8- (DiGeorge Critical Region 8; a double-stranded RNA binding protein involved in microRNA processing upstream of DICER) mutant spermatocytes display chromosome fusions (involving sex chromosomes), abnormal localization of proteins involved in telomere maintenance and increased accumulation of phosphorylated ATM in the sex body (Modzelewski et al., 2015).

The RNAi machinery is, in addition, involved in higher-order chromatin structure such as centromeres during mitosis and meiosis (Hall et al., 2003; Claycomb et al., 2009; Pek and Kai, 2011; Goto and Nakayama, 2012; Huang et al., 2015). For example, in *Argonaute* mutants of maize and rice, centromeric histones are mis-localized during meiosis, leading to chromosome segregation defects (Nonomura et al., 2007; Singh et al., 2011). In *S. pombe*, the formation and maintenance of heterochromatin at centromeres is facilitated by RNAi proteins and their partner, the histone methyl-transferase Clr4 (Zhang et al., 2008). Interestingly, the chromatin state, as controlled by the Dcr1/Clr4 pathway, can differentially regulate the accessibility of DNA to transcription or recombination proteins (Ellermeier et al., 2010). Indeed, while centromeric recombination is very low in wild-type fission yeast, as in most species, the *dcr1* and *clr4* mutants display increased meiotic recombination at centromeres, as assessed by recombining markers on each side of the centromere of chromosome 3 (Ellermeier et al., 2010). In contrast, recombination on chromosome 3 arms remains unchanged. Recombination increase is associated with increased Spo11-dependent double-strand break formation and/or their accumulation at centromeres in *dcr1*, but is independent of the repression of centromeric gene transcription (Ellermeier et al., 2010).

The above information implicates the RNAi machinery in the meiotic process, but leaves open the question of how these proteins impact the specific steps of prophase chromosome dynamics such as pairing, formation of the meiosis-specific structure called synaptonemal complex (SC), and homologous recombination. Here we exploited the power of *Sordaria macrospora* as a particularly attractive experimental system (Zickler and Espagne, 2016) for examination of the roles of both Dicer and Argonaute proteins during its sexual cycle. Initiation of meiosis and progression of nuclei through the various meiotic stages can be monitored independently of the chromosome status, by progressive increase in ascus/meiocyte sizes, thus permitting a clear establishment of events in mutant situations in comparison with wild-type meiosis. *S. macrospora* genome contains two Dicer-like homologs (*DCL1* and *DCL2*) and two Argonaute (*QDE2* and *SMS2*) genes. We show that the four single null mutants exhibit normal vegetative growth but significant meiotic defects, with different phenotypes in each mutant. While the *dcl2* and *qde2* mutants exhibit almost normal meiotic progression and sporulation, *dcl1* and *sms2* mutants display severe defects in the early stages of meiocyte formation. Moreover, when compared to wild type, *dcl2, qde2*, and especially *dcl1* mutants exhibit longer chromosome axes and synaptonemal complexes, plus higher crossover (CO) numbers with altered patterning and crossover interference. The *sms2* mutant displays, moreover, altered synaptonemal complex formation and E3-ligase Hei10 localization. Taken together, these findings indicate clearly that the RNAi components play a central role in the meiotic process of *Sordaria* and extend our understanding of the different processes (e.g., axis length, crossover patterning) that are directly or indirectly dependent on the Dicer and/or the Argonaute proteins. They reveal, moreover, a synergistic relationship between Dcl1 and Dcl2 as well as between Dcl1 and Sms2, with respects to some of these effects.

## Materials and Methods

### Identification of *S. macrospora* Dicer and Argonaute Genes

Dicer protein accession numbers used here are: *S. macrospora* Dcl1 SMAC_00946 and Dcl2 SMAC_06757; *Schizosaccharomyces pombe* Dcr1 Q09884; *Arabidopsis thaliana* DCL1 AEE27221, DCL2 AEE73925, DCL3 Q9LXW7, and DCL4 P84634; *Drosophila melanogaster* Dcr1 NP_524453.1 and Dcr2 NP_523778.2; *Caenorhabditis elegans* DCR-1 CE47418; and *Homo sapiens* Dicer AAI50288.1. Argonaute protein accession numbers are: *S. macrospora* Qde2 SMAC_03832 and Sms2 SMAC_08605*;* and *S. pombe* Ago1 NP_587782; *Arabidopsis thaliana* AGO2 NP_174413, AGO3 NP_174414, and AGO7 NP_177103*; Drosophila melanogaster* Ago1 NP_725341 and Ago2 ABO27430*; Caenorhabditis elegans* ALG-1 NP_510322 and ALG-2 NP_871992*;* and *Homo sapiens* AGO1 NP_036331, AGO2 NP_036286, AGO3 NP_079128, and AGO4 NP_060099. Most domains were identified using the NCBI database^1^. The divergent PAZ domains were identified using the Phyre2 fold recognition server^2^; Kelley et al., 2015). In both Dcl1 and Dcl2, a platform-paz-connector cassette was identified at position 591–804 and 749–1049, with 100 and 98% confidence, respectively. In *S. pombe*, the same cassette was predicted at position 663–894 with a 100% confidence. For Qde2, a PAZ domain was predicted by Phyre2 at position 369–570 (99.9% confidence). Illustrations were made using https://prosite.expasy.org/mydomains.

### RT-qPCR Experiments

Cultures for RNA preparations were performed on 90 mm Petri dishes containing M2 minimal medium and covered with a cellophane sheet (Focus Packaging and Design Ltd., Scunthorpe, United Kingdom). A total of 36 dishes (4 biological replicates, 9 days) were inoculated with one wild-type implant in the middle of the Petri dish. Dishes were placed at 25°C under constant light and four of the plates were removed from the incubation room every day from day one to nine. At day 1 and 2, the vegetative mycelium was scraped from the cellophane sheets with a glass cover-slip. From day 3 to day 9, the sexual cycle starts and the corresponding perithecia (fruiting bodies) were harvested by scraping them with a scalpel blade. RNAs were extracted with the RNeasy Plant Mini Kit (Qiagen, Hilden, Germany) according to supplier instructions. DNase digestions were performed in solution with RNase-free DNase Set (Qiagen, Hilden, Germany), followed by RNA cleanup on the RNeasy Plant columns as recommended by Qiagen. RNAs were quantified and checked for integrity on a gel. Total RNAs were reverse transcribed with SuperScript III (Invitrogen, Carlsbad, CA, United States) according to manufacturer’s instructions with oligo d(T)20. Primers used for RT-qPCR are shown in Supplementary Table 1. Most primer pairs were designed to ensure specific detection of cDNA, with at least one primer encompassing two consecutive exons. Specificity of these primers was checked experimentally during the design. In addition, a non-reverse-transcribed (NRT) control was performed on a pool of biological replicates to check the specificity of the detection. For primer pairs that are not specific for cDNAs, an NRT control was performed for each biological replicate to check the NRT-qPCR signal is low enough to allow a reliable analysis of the RT-qPCR signal (Supplementary File 1). Four biological replicates were performed for each day and each biological replicate was analyzed in technical duplicate. The sample maximization method (Hellemans et al., 2008) was used as the experimental set-up for plate design, which also included serial dilutions of a pool of biological replicates to compute amplification efficiency, NRT and negative controls. The average Cq for each gene in each biological replicate is shown in Supplementary File 1. Four reference genes (*PDF2, TIP, UBC*, and *CIT1*) were selected in a set of eight housekeeping genes using geNorm (Vandesompele et al., 2002). The average expression stability of these four genes is *M* = 0.542, indicating heterogeneous biological replicates (Hellemans and Vandesompele, 2014), and V4/5 = 0.121 which is below the cut-off recommended for geNorm (Supplementary File 1). Fold changes were computed with REST 2009 (Pfaffl et al., 2002). Fold changes were expressed relative to day 1. RT-qPCR experiments were MIQE compliant (Bustin et al., 2009). RNAseq data has been deposited under PRJEB44726.

### Mutants

Plasmids containing deletion cassettes conferring resistance to hygromycin B were constructed according to the *N*. *crassa* strategy for high-throughput generation of gene deletion (Colot et al., 2006) with modifications aimed at minimizing errors in the 5′ and 3′ flanking regions, notably by the use of Phusion DNA polymerase (Thermo Scientific) (see Supplementary Table 2 for primer sequences). The deletion cassettes were released from the vector by *Asc*I digestion prior to transformation. The structure of each transformant was confirmed by PCR analysis and DNA sequencing (Genewiz). Transformations were performed in a *ku70* mutant background, which increases the homologous integration events. Further crosses with a Ku70 strain eliminated the *ku70* allele. The entire ORF is deleted in the *dcl1, dcl2*, and *qde2* null mutants. Subsequent analysis of *SMS2* revealed that the deletion in the *sms2* mutant left 165 bp (55 amino acids) in 5′ of the CDS. No conserved domains are found in these 55 amino acids. We also performed the complete deletion of the *SMS2* ORF, and both mutants show the same defects in term of ascus formation and sporulation.

### Cytology

Ascus-bouquet pictures were taken on living material and imaged with transmitted light. GFP and mCherry fusion proteins, as well as DAPI (0.5 μg/mL) signals were observed, after fixation in 4% paraformaldehyde, with a Zeiss Axioplan microscope connected to a CCD Princeton camera or a Leica DMIRE2 microscope with a CoolSNAPHQ CCD camera (Roper Scientific). MetaMorph software (Universal Imaging Corp.) and public domain software ImageJ^3^ were used to deconvolute Z-series and treat the images. Images for the *dcl1* mutant were pooled from two different strains: *dcl1 ku70* Spo76-GFP Hei10-GFP and *dcl1* Spo76-GFP Hei10-mCherry. No differences in axis lengths or Hei10 foci numbers were detected between the two strains. All the other mutants were analyzed in a Ku70 background and with the Spo76-GFP Hei10-mCherry combination of markers. To define crossover positions, we used the line tracing tool of Image J software to measure the distances between the Hei10 foci along each SC (each trace was initiated at the center of the GFP or mCherry focus).

### Assessment of Vegetative and Sexual Phenotypes

To evaluate vegetative growth, wild type, single and double mutant strains were inoculated onto M2 minimum-medium plates and incubated for four days at 25°C until coverage of the plates (Supplementary Figure 2A). Five plates were used for each strain and the mycelium growth, measured twice a day, was compared to wild-type growth. Development of protoperithecia and mature perithecia was monitored for over ten days. In strains where perithecia formed but no ascospores could be recovered on the lid, the perithecia were cracked open using forceps and their ascus and ascospore formation was evaluated under dissecting microscope.

### Crossover Interference Analyses

Crossover interference was analyzed by two different methods: Coefficient of Coincidence (CoC) and gamma distribution. The CoC (the classical way to define CO interference; Muller, 1916) directly represents the extent to which COs in two different intervals (defined genetically or along the SC) do or do not occur independently. Therefore, in our analysis, the SC of each chromosome is first divided into a number of intervals of equal size. To be sure that all closely spaced COs are counted, the number of intervals must be sufficiently high. A general rule is that the interval size should be less than 1/4 of the average distance between COs (Zhang et al., 2014a). Along each SC of the data set (around 200 SCs for each analyzed mutant), each CO/Hei10 focus is thus assigned to a specific interval along each SC of the data set. We can then assess, for any given pair of intervals, the number of SCs containing a CO in both intervals (double COs) within our dataset. The frequency of double COs is then examined and compared to the frequency of the expected double COs if they occurred independently in the same two intervals (i.e., in the two intervals considered individually). The CoC is the quotient of observed/expected double COs. The same comparison is done for all possible pairs of intervals. The CoCs of all pairs of intervals are further plotted, for each pair, as a function of the distances between two intervals (defined as the distance between the centers of the two intervals): this gives the CoC curve. In our analyses, the CoC patterns were calculated and grouped for the two longest chromosomes (1 and 2) and for the other five smaller chromosomes. The CoC values are very low for short inter-interval distances, reflecting the fact that CO interference is stronger at short distances. They increase with increasing inter-interval distance to a value of one, which indicates independent occurrence in the two intervals. The fluctuation around and above one corresponds to the tendency for even spacing of the COs. Gamma distribution analysis is another method used for describing interference. For this purpose, the inter-adjacent Hei10 foci distances in wild type and mutants are calculated as percentages of physical distances (μm). The best-fit gamma parameters for all inter-Hei10 foci distances along all chromosomes were estimated by the maximum likelihood method with the “gamfit” function in MATLAB.

### Statistical Analyses

All analyses were carried out using one-way ANOVA and non-parametric tests on GraphPad/Prism. All values are given as mean ± standard deviation (SD). For axis length comparisons, a Brown-Forsythe ANOVA test for the equality of group variance was performed and showed significant differences [F (3, 72.98) = 103.8, *P* < 0.0001] followed by a *post hoc* Games-Howell test (reported in the Figures). For the foci number comparison, the Brown-Forsythe ANOVA also showed significant differences [F (3, 96.21) = 79.92, *P* < 0.0001] followed by a *post hoc* Dunnett’s T3 test.

## Results

### *Sordaria macrospora* Genome Encodes Two Dicer-Like and Two Argonaute Proteins

A BLAST protein homology search using *N. crassa* protein sequence as reference revealed that the genome of *S. macrospora* encodes two Dicer homologs, hereafter referred to as Dcl1 and Dcl2, as well as two Argonaute homologs, named after the *N. crassa* nomenclature Qde2 and Sms2. Each gene annotation has been checked using our own RNAseq data (unpublished). While the *DCL1*, *DCL2* and *QDE2* annotations obtained from this comparison are correct, analysis of *SMS2* transcripts revealed that two small extra exons are present in 5′ of the gene. The new coding sequence obtained after correction is extended by 165 bp (55 amino acids) at 5′ of the gene and is identical to the gene present in the new version of the *S. macrospora* genome (Blank-Landeshammer et al., 2019). No conserved domains were found in the added 55 amino acids. Domain conservation searches uncovered most of the known functional domains, including a PAZ (Piwi Argonaute Zwille) domain (identified either through NCBI Conserved Domain database or by structural homology using the Phyre2 software, see section “Materials and Methods” and Supplementary Figure 1A).

Based on RT-qPCR in wild type, all four genes are expressed during the sexual cycle and the meiotic divisions, with temporal kinetics that exactly parallel those of *SPO11*, which encodes the meiotic transesterase that promotes double-strand break (DSB) formation, and with *MER2*, which encodes a protein that mediates assembly of recombination-initiation complexes and DSBs (Supplementary Figure 1B). *SMS2* shows the strongest upregulation with more than a 2,000-fold increase compared to the vegetative state (day 1). *DCL1* transcript abundance is increased 8- to 16-fold, while *DCL2* and *QDE2* transcript levels are increased only 3-fold during the sexual cycle. These patterns are similar to those obtained in the fungus *F. graminearum*: while *FgDICER1* and *FgAGO2/FgSMS-2* were strongly upregulated during the sexual cycle, *FgDICER2* transcript levels were maintained throughout the developmental stages, and *FgAGO1* showed only a moderate increase during fruiting-body formation (Son et al., 2017).

### The Four Proteins Are Not Required for Vegetative Growth but Dcl1 and Sms2 Are Essential for Correct Ascus/Meiocyte Formation and Sporulation

Comparison of hyphal (vegetative cells) extension rate in wild type, the four null mutants and the *dcl1 sms2* and *dcl1 dcl2* double mutants, shows that none of the four RNAi components are required for the fungal vegetative growth (Supplementary Figures 2A,B).

**FIGURE 1:**
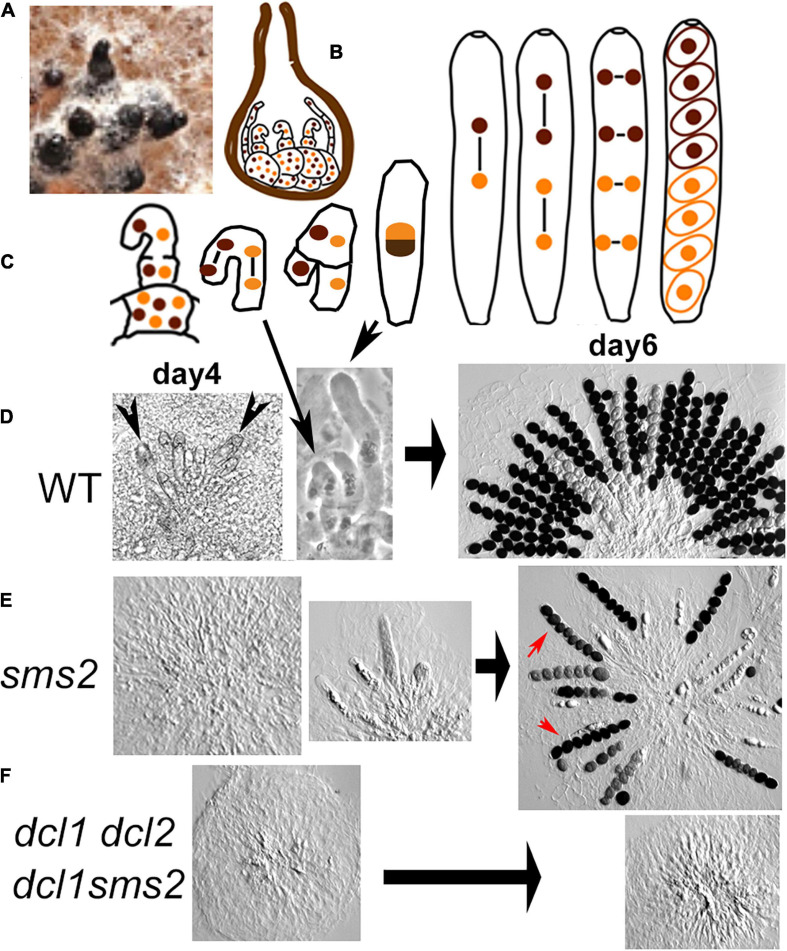
Meiocyte formation and sporulation in wild type, *dcl1*, and double mutants. **(A)** Six bottle-shaped perithecia (black) developed on growing mycelium (white) on agar medium (brown). **(B)** Drawing of a section through a perithecium in which binucleated cells (middle) and paraphyses (two on both sides) have started to develop from the ascogonium multinuclear cells (at the base). **(C)** Progression from crozier to ascus formation, followed by karyogamy, the two meiotic divisions, a post-meiotic division and ascospore delimitation in wild type (WT). **(D–F)** Light micrographs of asci dissected out from perithecia. **(D)** Left and middle: WT young perithecia contain several small asci (arrowheads, left), intermingled with croziers (long arrow, center); right: mature perithecium with eight-spored asci (half of the asci presented here). **(E)** Left, middle and right: corresponding *sms2* stages. Note the reduced number of asci when compared to WT, the small number of asci with eight regularly shaped ascospores (red arrows) and the presence of abnormal, often aborted ascospores in the other asci. **(F)** Perithecia of the two double mutants contain paraphyses but no asci even after 6 and 14 days.

Three of the mutants are, in contrast, defective in the complex multicellular differentiation processes occurring during the sexual cycle. This cycle initiates with the development of an aggregation of hyphae around the female ascogonia: the protoperithecia. This early step is completely wild-type (in number on the plates and in timing) in the four single and the two double-mutant strains. Further growth and expansion of the protoperithecium into a flask-shaped perithecium (called also fruiting body; Figure 1A) is accompanied by differentiation of several cell types inside this structure. (i) The multinuclear ascogonium differentiates into hyphae/cells that now contain only two nuclei, called ascogenous hyphae, and gets surrounded by several hyphae called paraphyses (Figure 1B). (ii) After a few synchronous divisions, the tip cells of the ascogenous hyphae differentiate into hook-shaped cells, the croziers (Figure 1C left), in which the two nuclei undergo one coordinated mitosis yielding, after septal formation, two uninucleate basal cells and a binucleate ascus mother cell (Figure 1C and long arrow in Figure 1D). (iii) Karyogamy and meiosis occur in the ascus mother cell and after a post-meiotic mitosis, eight ascospores are delineated in this single cell (Figure 1C from middle to right). (iv) Each wild-type perithecium contains several croziers and young asci (arrowheads in Figure 1D left) and finally over 100 eight-spored asci/meiocytes (Figure 1D right).

Protoperithecia develop into flask-shaped perithecia in all six mutant strains, but the differentiation of the ascogenous hyphae into several asci and finally eight-spored asci inside the perithecium (Figure 1D left through right) is only wild-type-like in the *dcl2* and *qde2* null mutants (Supplementary Figure 3A). In contrast, *dcl1* and *sms2* null mutants display both delayed and strongly defective ascus formation (illustrated for *sms2* in Figure 1E left and middle and in Supplementary Figure 3B for *dcl1*). After 5 days post-inoculation, *dcl2* and *qde2* perithecia contain over 100 eight-spored asci (Supplementary Figure 3A), like wild type (Figure 1D right). In contrast, only 6% of *dcl1* and 42% of the *sms2* perithecia (≥200 crack-opened for each strain) contain asci and always in highly reduced numbers (1 to 10; illustrated in Figure 1E middle and right plus Supplementary Figure 3C for *sms2* and in Supplementary Figure 3B for *dcl1*), the remaining perithecia containing only paraphyses (Figure 1E left). On the sixth day, all *sms2* perithecia contain 5 to 30 asci (Supplementary Figure 3C) but 80% of the *dcl1* perithecia still contain only paraphyses (*n* = 200 for each strain).

These two mutants exhibit two more intriguing phenotypes: (i) while all perithecia of the wild-type plates contain over 100 asci, *dcl1* and *sms2* plates contain always a mixture of perithecia with various numbers of asci (compare Figure 1D right with Figure 1E right); (ii) furthermore, the phenotypes of the asci inside each perithecium are also variable: a few asci exhibit eight regularly formed ascospores (red arrows in Figure 1E right) when the other asci contain abnormal ascospores in number and shape (all other asci with spores in Figure 1E right).

In order to decipher potential epistasis between the different mutations, we generated all six double mutants. First, no spores are observed in the *dcl1 dcl2* and *dcl1 sms2* double mutants even after 10 days post-inoculation (0/205 cracked-open perithecia Figure 1F and Supplementary Figure 2C). This suggests that Dcl1 and Sms2 are both required, in a non-redundant, non-epistatic fashion, for ascus/meiocyte formation, and sporulation. Moreover, when both Dicer proteins (in the *dcl1 dcl2* double mutant) or both Argonaute proteins (in the *qde2 sms2* double mutant) are absent, virtually no asci and spores are formed (asci only in 3 or 0% of the perithecia, *n* = 203 and 205, respectively). This indicates that at least one protein of each functional group is strictly required for proper ascus/meiocyte induction and sporulation in *Sordaria*. This also points out that, while both *dcl2* and *qde2* single mutants show wild-type levels of sporulation (Supplementary Figure 3A), both Dcl2 and Qde2 are nevertheless implicated, albeit to a lesser extent (but which becomes important in absence of Dcl1 or Sms2), in the meiocyte/sporulation process.

The other double mutants did not rescue or worsen the sporulation phenotypes of the corresponding single mutants: (i) *dcl1 qde2* and *sms2 dcl2* double mutants (Supplementary Figure 3D left and middle) show the same sporulation defects as *dcl1* and *sms2* single mutants, respectively (Supplementary Figures 3B,C); (ii) the *qde2 dcl2* double mutant displays wild-type levels of sporulation (Supplementary Figure 3D right). These results show that the RNAi pathway is required for proper ascus/meiocyte induction and sporulation in *Sordaria*, with prominent roles for Dcl1 and Sms2, and a lesser role for Dcl2 and Qde2.

### During Meiotic Prophase, Dcl1, Dcl2, Sms2, and Qde2 Are Not Required for Axis and Synaptonemal Complex Formation but Dcl1 and Sms2 Are Essential for Their Maintenance

The four null mutants progress through meiosis with the same gradual increase of ascus size as in wild type, allowing meiotic prophase analysis without complications from defects in ascus growth. It should be noted that contrary to the *dcl2* and *qde2* mutants which produce large numbers of prophase asci per perithecium, the effects of *dcl1* and *sms2* deletions on meiotic prophase could only be assessed in the 1 to 30 asci/meiocytes produced per perithecium. Chromosomes were analyzed in leptotene through pachytene nuclei by DAPI staining of the chromatin and with the previously characterized axis marker Spo76/Pds5 (Van Heemst et al., 1999).

In wild-type *Sordaria*, chromosome axes develop at early leptotene with accompanying initiation of recombination by the induction of DSBs by Spo11 (Storlazzi et al., 2003). Repair of these DSBs by homologous recombination then allows recognition and spatial juxtaposition of the homologous chromosomes (homologs) at a distance of around 400 nm through robust inter-axis bridges (Dubois et al., 2019). Synapsis then occurs, bringing homologous axes together at a distance of 100 nm via formation of the synaptonemal complex (SC) along the seven homologs (Espagne et al., 2011). The latter steps of recombination occur in the context of the SC (Tessé et al., 2017). A similar chromosomal progression is observed in the four mutants, except that the synaptic period is slightly prolonged in *dcl2* (by 12 h) and even more greatly delayed in *dcl1* and *sms2* (by over 24 h).

**FIGURE 2:**
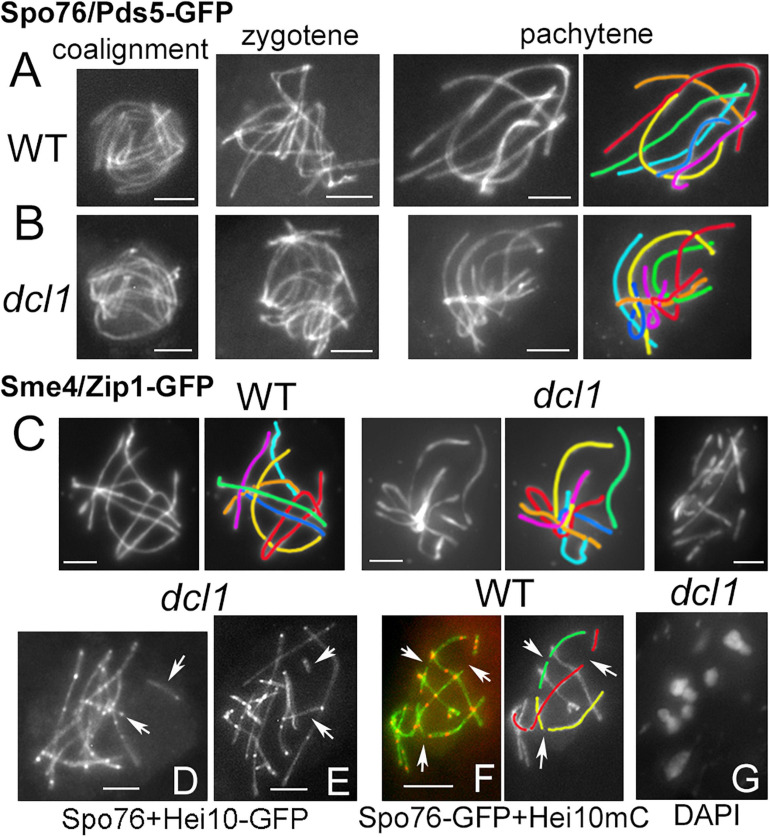
Synaptonemal complex in wild type and *dcl1*. **(A,B)** Chromosome axes are marked by Spo76/Pds5-GFP. From left to right: progression from coalignment at late leptotene, partial synapsis at zygotene (middle) and complete synapsis at pachytene in wild type **(A)** and *dcl1* **(B)**; the seven homologs are distinguishable by their lengths and colors. **(C)** SCs are marked by the central component Sme4/Zip1-GFP in wild type (left) and in *dcl1* with full SC along all homologs (middle). Right: example of a *dcl1* pachytene nucleus with more than 7 SCs. **(D,E)** Two *dcl1* nuclei with extra SC segments (arrows), either separated from the other SCs (upper arrows), or at odd angles (lower arrows). **(F)** Interrupted SCs are also seen in WT late pachytene nuclei (arrows), but the SC paths can be followed in the concerned homologs (illustrated for three of them, right). **(G)** Diplotene of *dcl1* with 7 bivalents. Bars, 2 μm.

Like in wild type (Figure 2A left), Spo76/Pds5 forms complete lines along chromosomes at leptotene in all four mutants (illustrated for *dcl1* in Figure 2B left), indicating that Dicer and Argonaute proteins are not required for chromosome axis formation during onset of meiosis. The four mutants have also no problem with forming SC continuously along the seven *Sordaria* homologs as tested by observation of their axes (by Spo76/Pds5-GFP), which are now at 100 nm distance and form a single line (compare Figure 2B with Figure 2A; and see below for the other mutants) and by labeling the SC central component Sme4/Zip1 (Figure 2C).

After mid-pachytene (by ascus size), the *dcl1* and *sms2* mutants exhibit a defect in SC maintenance. In *dcl1*, the SC is initially complete from telomere to telomere at early pachytene (Figures 2B,C middle). However, among the 29 analyzed mid-late pachytene *dcl1* nuclei, nine exhibit more than seven segments corresponding to the seven pairs of homologs seen at early pachytene. These nuclei contain 8 to 13 SC segments observed either by Sme4/Zip1-GFP (Figure 2C right) or by Spo76/Pds5-GFP (arrows in Figures 2D,E). The SC segments have variable lengths (from 1 to 4 μm), but are always shorter than the shorter *dcl1* chromosome (7.3 ± 1.1 μm). Nevertheless, total axis/SC lengths in these nine nuclei are similar to the total axis lengths measured in the other 20 nuclei that show seven uninterrupted SCs (79.3 ± 12.8 compared to 73.4 ± 8.9 μm), suggesting early SC disassembly in those nuclei and not SC formation between sister chromatids as observed in haploid meiosis of *Sordaria* (Vasnier et al., 2014) or in absence of Rec8 in mouse (Xu et al., 2005).

In some respect, this *dcl1* mutant phenotype resembles the pattern seen in wild type when the SC components start to disassemble at late pachytene, at which point the nuclei exhibit also more than seven SCs segments (arrows in Figure 2F). However, in the wild-type case, one can easily follow the paths through the “missing” segments and thus “reconstruct” the original seven SCs (illustrated by the red, yellow, and green homologs in Figure 2F). In contrast, in *dcl1* the extra short SC segments are at odd angles to the “regular,” longer, SC segments and/or very far from them (arrows in Figures 2E,F). Unfortunately, at late pachytene, chromatin is too diffuse in *dcl1* to infer connections between the smaller segments of SCs to one or the other of the longer SC segments. These data point to a defect in SC maintenance in the *dcl1* mutant. In accord with this interpretation, we failed to detect broken chromosomes in the 12 diplotene nuclei analyzed (Figure 2G), implying that the defect observed at mid-late pachytene represents aberrant discontinuity only at the SC structural level but it remains unclear why the SC segments have lost their continuity.

**FIGURE 3:**
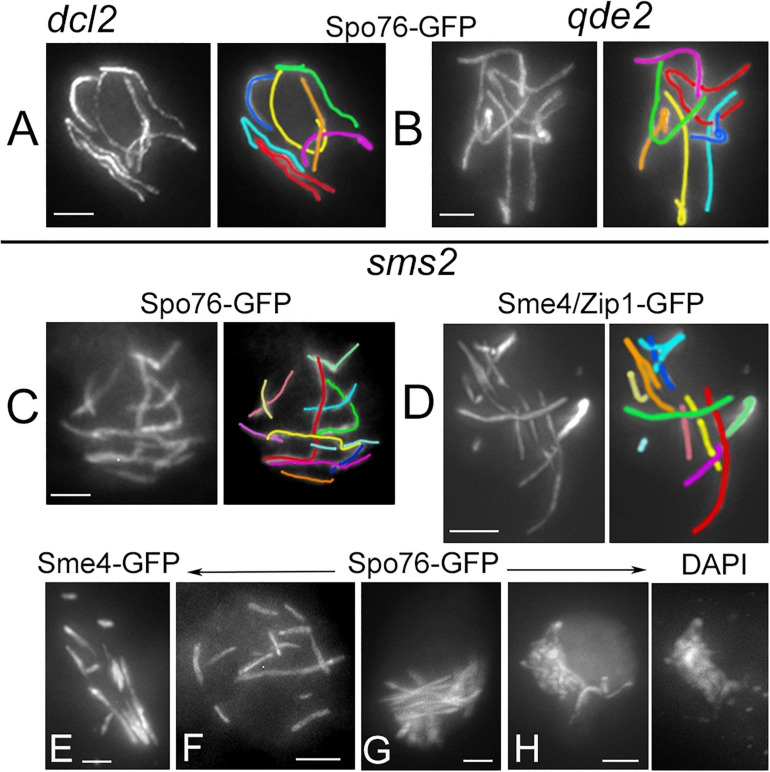
Defective SC formation in *sms2* when compared with *dcl2* and *qde2*. **(A–H)** SCs are marked by Spo76/Pds5-GFP or Sme4/Zip1-GFP. **(A,B)** SCs extend from telomere to telomere in pachytene nuclei of *dcl2* **(A)** and *qde2* **(B)**; the seven homologs are distinguishable by their lengths and colors. **(C–H)** Examples of abnormal SCs in *sms2* pachytene nuclei. **(C,D)** Two examples with 12 and 11 SC segments in mid-pachytene nuclei (by ascus size), and corresponding colors. **(E,F)** SCs are either stiff and close **(E)** or widely separated **(F)**. **(G)** Example of stiff and intermingled SCs. **(H)** Late pachytene nucleus with highly intermingled SCs and, right, corresponding DAPI. Bars, 2 μm.

The tendency for aberrant and variable numbers of SC segments is even more pronounced in absence of Sms2. When all pachytene nuclei of *dcl2* and *qde2* (*n* = 37 and 43, respectively) show seven continuous Spo76/Pds5-GFP lines when the homologous axes are synapsed at 100 nm distance by the SC (Figures 3A,B), none of the *sms2* analyzed nuclei (*n* = 37) exhibit such continuous lines. During what should be early to mid-pachytene by ascus size, the *sms2* nuclei marked by either Spo76-GFP (Figure 3C) or by Sme4/Zip1-GFP (Figure 3D, *n* = 10) exhibit a total of 8 to 14 SC segments. These SC segments are, moreover, aberrant in their overall disposition in various ways: either stiff and close (Figure 3E) or largely separated from one another (Figure 3F) or completely intermingled (Figure 3G). Finally, at what should be late pachytene by ascus size, SC segments are even more intermingled (Figure 3H) and chromatin is very fuzzy (Figure 3H right), indication that those nuclei are likely degenerating, in accordance with the fact that this mutant shows also a high number of abnormal asci and spores as described above (Supplementary Figure 3C). Both *dcl1* and *sms2* SC phenotypes, and the presence of diffuse chromatin (Figure 3H), suggest that the observed SC discontinuities could result from local changes in the chromatin structure, which could alter SC integrity, leading to aberrant or premature local SC disassembly.

### Dcl1, Dlc2, and Qde2 Are Required for Axis/SC Lengths and Crossover Numbers

**FIGURE 4:**
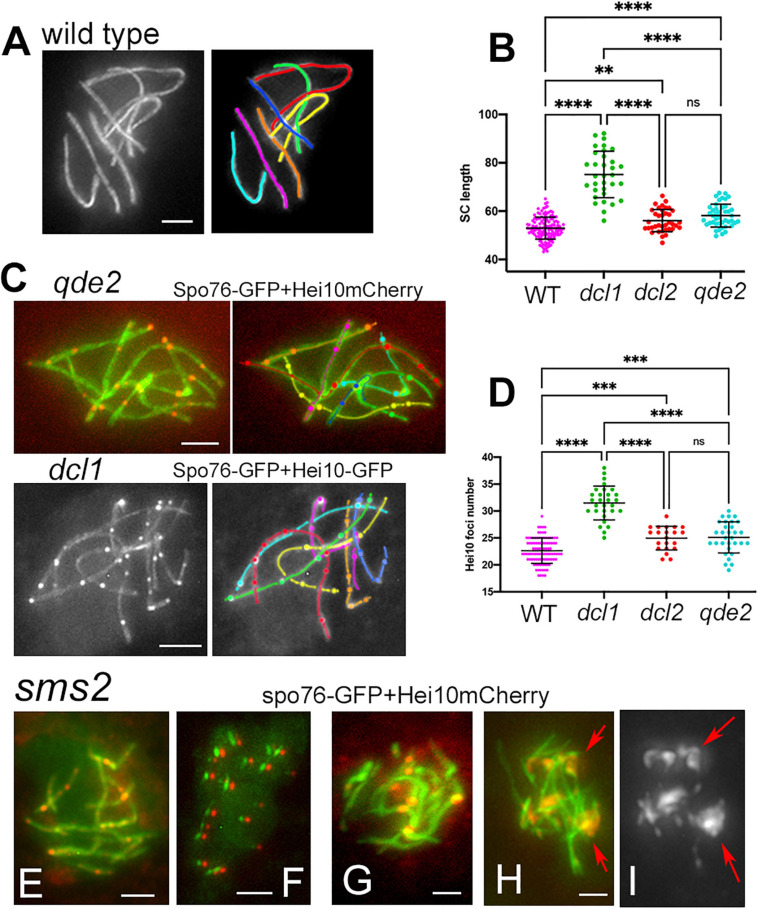
Axis lengths and crossover numbers in WT, *dcl1, dcl2*, and *qde2* mutants plus Hei10 foci in *sms2*. **(A)** Wild-type pachytene nucleus with 7 Spo76-GFP marked SCs. **(B)** SC lengths per nucleus in WT and mutants; means with standard deviations; *P*-values: ^∗∗^*p* < 0.01; ^****^p < 0.001. **(C)** Examples of Hei10 foci localization along the seven homologs of *qde2* (top) and *dcl1* (bottom). Axes are marked by Spo76-GFP and foci are marked either by Hei10-mCherry (*qde2*) or by Hei10-GFP (*dcl1*). **(D)** Number of Hei10 foci per nucleus in WT and mutants; double lines indicate standard deviations; *P*-values: ^∗∗^*p* < 0.01; ^****^p < 0.001. **(E–I)** *sms2* mutant. **(E)** Nucleus with extra SCs (see Figure 3C) but wild-type like Hei10 foci [compare with the *qde2* nucleus in **(C)**]. **(F)** At early diffuse stage, only small SC segments (green) are left but they contain all one Hei10 focus (red). Green and red signals from SC and foci, respectively, were shifted for easier observation. **(G)** SCs are abnormal in length and shape and foci are larger than normal [compare with **(E)**]. **(H,I)** Nucleus with large Hei10 foci with **(H)** and without **(I)** Spo76-GFP. Foci are either single or aggregated [red arrows in **(I)**]. Bars, 2 μm.

To assess the possible role of the RNAi components in axis and/or SC lengths, we analyzed SC lengths in wild type and in the three mutants *dcl1*, *dcl2*, and *qde2* that exhibit seven bivalents throughout pachytene (Figures 4A,B). The most prominent increase in SC length is observed in the *dcl1* mutant: 75.2 ± 9 μm (*n* = 33 nuclei) compared to 52.9 ± 4.5 in wild type (*n* = 130). Although less pronounced, the mean SC lengths of the *dcl2* (56.1 ± 4.6; *n* = 37) and *qde2* (58.1 ± 4.7; *n* = 43) mutants are also significantly different from wild-type lengths but are not significantly different from one another (Anova tests; Figure 4B). All these increased lengths are, moreover, not specific to the SC stage because Spo76-labeled chromosome axes are already longer than wild-type axes at the coalignment and early zygotene stages (e.g., for *dcl1*, Figure 2B left; 76.3 ± 8.2, *n* = 10).

Crossover numbers were defined by cytological analysis of E3-ligase Hei10 foci, which mark the sites of CO-fated recombinational interactions in pachytene nuclei (De Muyt et al., 2014) when SC is full length (illustrated in Figure 4C for *qde2* and *dcl1*). The mean number of Hei10 foci per pachytene nucleus is, respectively, of 31.5 ± 3.1 in *dcl1* (*n* = 29 nuclei), 24.9 ± 2.2 in *dcl2* (*n* = 21) and 25.1 ± 2.9 in *qde2* (*n* = 31) compared to 22.6 ± 2.3 in wild type (*n* = 98 nuclei). Thus, paralleling the increase in SC lengths, there are significantly more Hei10 foci per nucleus, and thus COs, in the three mutant strains than in wild type; and the increases observed in *dcl2* and *qde2* are not significantly different from one another (Figure 4D).

The distribution of the Hei10 foci in the mutants exhibit also four hallmarks of meiotic COs. (i) All bivalents of *dcl1*, *dcl2*, and *qde2* (567 in total) show at least one Hei10 focus, indicating the presence of the “obligatory crossover” (required for proper chromosome segregation) in all three mutants. (ii) Like in wild type, the total number of CO events varies appreciably from one nucleus to another (Figure 4D). (iii) Within the nuclei with high numbers of Hei10 foci, all seven chromosomes exhibit correlated increased numbers of COs, showing that increases are global and not chromosome specific. (iv) Linear regression analysis shows that in each nucleus, SC length and CO number are correlated (Supplementary Figure 4A).

The number and position of pachytene Hei10 foci reflect the nature of the CO-designation/interference process that occurs at earlier stages and could thus be sensitive to defective numbers of DSBs. To investigate this possibility, we measured the numbers of Mer3 foci that initially occur, in the same number as Rad51 foci, and mark the sites of DSBs in *Sordaria* (Storlazzi et al., 2010). In the *dcl1* mutant, with the highest number of COs, the number of Mer3 foci is similar to wild type: 54.2 ± 14.7 (*n* = 14) vs. 61.9 ± 22 (*n* = 24, *p* = 0.3) (Supplementary Figure 4B). Therefore, we infer that the increase in CO number does not stem from an increase in DSB formation, but rather from a deregulation of downstream events.

In the *sms2* mutant, despite the very defective axis organization and SC formation and/or elongation at pachytene (see above), Hei10 foci form in all pachytene nuclei (Figure 4E). Among the 27 pachytene nuclei analyzed, 14 showed 20.3 ± 6.9 Hei10 foci, thus close to the number of 22.6 ± 2.3 foci observed in wild type (compare Figures 4E–G). Also, like in wild type, those foci persist through the early diffuse stage where they remain associated with a residual stretch of SC (Figure 4F). The foci in these 14 nuclei exhibit, moreover, the same size and fluorescence density as those of wild type and the three other mutants (Figures 4E,F). The 13 other nuclei, in contrast, show very intriguing phenotypes: instead of being single, Hei10 forms more or less large bright masses (Figures 4G,H) where two to several foci are stacked (arrows in Figure 4I). To our knowledge, this is the first meiotic mutant to display such a phenotype. Whether these masses come from the aggregation of multiple foci at different loci, or from accumulation of Hei10 protein at individual recombination sites remains unknown.

### Crossover Interference and Patterning Is Perturbed in *dcl1*, and to a Lesser Extent in the *dcl2* and *qde2* Mutants

Crossover patterns arise in two stages during meiotic prophase. Recombination is first initiated by a large number of programmed DSBs which occur in recombination complexes that are associated with the chromosome axes. A small subset of those DSBs are then specifically designated to become COs. Finally, CO-designated interactions mature to actual COs, in association with the SC components, via a series of further biochemical steps. Several mechanisms limit the number of precursors that will effectively be turned into a CO, channeling the other DSBs to being repaired as non-crossovers (e.g., Hunter, 2015; Lam and Keeney, 2015). One of these mechanisms, termed CO interference, was identified by the finding that a CO at one position disfavors occurrence of additional COs nearby, resulting in a tendency for COs to be more widely and regularly spaced than predicted from a random distribution [Muller, 1916; reviewed in Wang et al. (2015)].

**FIGURE 5:**
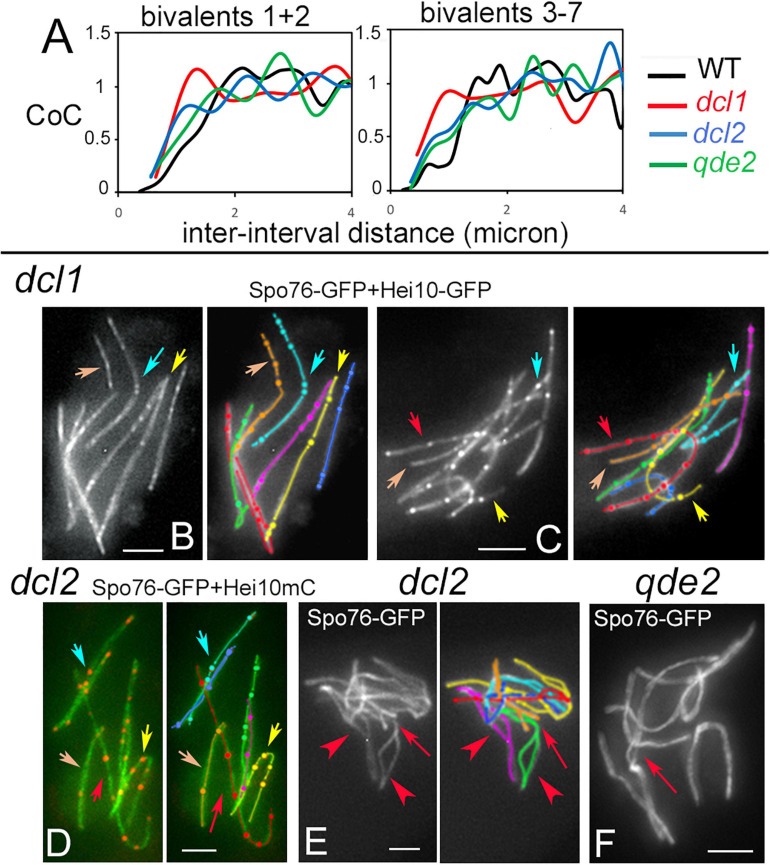
Crossover interference, patterns and synapsis defects in *dcl1*, *dcl2*, and *qde2*. **(A)** Coefficient of Coincidence (CoC) plotted as a function of inter-interval distances in longer homologs 1 and 2 (left) and shorter homologs 3 to 7 (right) of WT, *dcl1*, *dcl2*, and *qde2*. **(B,C)** Hei10 foci along homologs in *dcl1* early **(B)** and mid-pachytene **(C)** nuclei. Note that foci are brighter at the later stage **(C)**. Similar irregularities in focus distances are seen in *dcl1* (arrows in **B,C)** and *dcl2* **(D**, arrows). Only 4 of the 7 homologs are pointed by arrows for easy lecture, but the other homologs (e.g., the purple and green ones) show also variable distances and close foci. **(E)** Zygotene nucleus of *dcl2*. Note the widely open non-synapsed regions (arrowheads on green and purple homologs) and the resolving entanglement (arrow) between the green and orange homologs (open ends of the orange homolog will help the green homolog to slide out of the interlock). **(F)** Illustration of a non-resolved interlock at mid-late pachytene (by ascus size) in *qde2* (arrow). Bars, 2 μm.

The tendency for COs to be evenly spaced along the chromosomes, is reflected in the fact that the distribution of inter-CO distances along each homolog has the shape of a gamma distribution, with a value for the shape parameter ν greater than 1. Being a measure of evenness, the shape parameter ν of the gamma distribution is thus an indirect indicator of interference (see section “Materials and Methods”). In all three mutants, ν is decreased compared to wild type: 2.40 ± 0.13 for *dcl1*, 4.01 ± 0.27 for *dcl2* and 4.28 ± 0.24 for *qde2* compared to 5.03 ± 0.18 for wild-type. These values suggest that interference is present in the three mutants but is less robust than in wild type. Reduced interference in the mutants is further confirmed by the Coefficient of Coincidence (CoC) analysis (see details in section “Materials and Methods”). For this approach, the 203, 147, and 217 SC-labeled chromosomes of, respectively, *dcl1*, *dcl2*, and *qde2* were divided into multiple intervals. For every possible pair of intervals, the frequency of chromosomes with a CO in both intervals is compared with the frequency expected if COs occurred independently (observed double COs/expected double COs). The resulting ratios are plotted as a function of inter-interval distances (Figure 5A). There are significantly higher levels of double COs at shorter inter-interval distances in the mutants than in wild type, and double COs frequency increases as inter-interval distance increases, with finally CoC values fluctuating around one as in wild type, which indicates that there is no interference at this distance (Figure 5A). CoC patterns are similar for both long (1 and 2) and short (3 to 7) chromosomes (Figure 5A right and left). However, when compared to wild type, the three mutant CoC curves are shifted to the left, indicating defective interference. The greatest effect is observed for *dcl1* (Figure 5A). In principle, since CO number scales directly with axis/SC length in the mutants as in wild type (Supplementary Figure 4A) and in most organisms (Wang et al., 2019), one could expect that gamma and CoC values would be the same in the *dcl1*, *dcl2*, and *qde2* mutants. The reduced CO interference observed in the three mutants implies, therefore, that COs tend to be closer together or less regularly spaced than in wild type. We showed previously that reduction of CO interference in the *mer2–17* allele of *Sordaria* was due to the presence of a high number of very close Hei10 foci (Tessé et al., 2017). Detailed inspection of Hei10 focus localization along the SCs of *dcl1*, *dcl2*, and *qde2* reveals that the mutants exhibit also close foci at inter-focus distance of 0.1 to 0.4 μm, but as can be seen in the nuclei of Figures 4C, 5B–D, they are not the majority. We suspect, therefore, that the decrease of CO interference could result from the fact that the Hei10 foci (and thus COs) are distributed in a very irregular way along the homologs. Indeed, as illustrated in Figures 5B–D, the distances between Hei10 foci are highly irregular along most of the homologs. For example, when red-labeled homologs of *dcl1* and *dcl2* show regularly spaced foci (red arrows in Figures 5C,D), yellow-, cyan- and orange-labeled homologs in Figures 5B–D (marked by corresponding color arrows) exhibit highly irregular focus distances. There is, however, no chromosome effect, because in other nuclei, the “red homologs” show also irregular focus distances.

Crossover patterning defects may be related to the fact that, even though SC forms all along the homologs at pachytene, the three mutants also exhibit pairing and synapsis defects at earlier stages. First, both coalignment and synapsis are delayed (above). Second, contrary to wild-type zygotene, SC formation is asynchronous, with some bivalents still only half synapsed when the others are fully synapsed (Supplementary Figure 4C). In addition, zygotene nuclei exhibit both entanglement(s) and largely non-synapsed regions in one or two of the seven homologs (arrow and arrowheads in Figure 5E, see Supplementary Figures 4C,D for more examples). Third, while such entanglements are all resolved at early pachytene in wild type (Storlazzi et al., 2010), they persist throughout pachytene in all three mutants, even in *qde2* which has the least severe defects in SC formation (arrow in Figure 5F). In accordance with the more severe CO patterning defects, *dcl1* exhibits also the more severe pairing defects, in accord with a direct relationship between these two features. Notably, these phenotypes identify a Dcl1, Dcl2, and Qde2 sub-function specific to the pairing/synapsis process.

## Discussion

The current study provides novel insights concerning the role of the Dicer-like and Argonaute proteins. First, none of the four proteins is required for normal vegetative growth, while all have important roles in several aspects of the sexual cycle. Second, observations of meiotic prophase reveal that the four proteins, each in a different way, exert effects on determination of chromosome-axis length, and crossover patterning. Taken together, our results provide new perspectives from which to consider roles of these RNAi factors.

### New Roles for Dicer and Argonaute Proteins in the Early Steps of Meiocyte Differentiation

A complex multicellular differentiation process leads to the formation of asci/meiocyte during the sexual cycle of *Sordaria*. The two Dicer proteins and the Argonaute Sms2 protein are required for several stages of this process. The first defects detectable in the mutants occur during the transition from the multinuclear cell stage, which is prevalent during the vegetative cycle, to the stage when cells contain only two nuclei in the so-called dikaryon stage, which is a prerequisite for karyogamy and meiosis. This transition into the sexual cycle involves considerable cell differentiation (Figure 1, above) associated with drastic changes of the transcriptional program (Blank-Landeshammer et al., 2019). It is interesting to note that, analogously, deletion of Dicer and Argonaute homologs lead to embryonic death and/or defects in cell-type differentiation in mouse, worm, *Drosophila*, maize, and *A. thaliana* [reviewed in Gutbrod and Martienssen (2020)]. This analogy is particularly striking in light of the fact that these organisms are present in very divergent branches of eukaryotes.

Diverse roles for the Dicer and Argonaute proteins are identified at the next stages of meiocyte/ascus differentiation. With respect to the Dicer proteins, Dcl2 is dispensable for ascus formation and development; in contrast, Dcl1 is strictly required for both the vegetative to the sexual cycle transition and for wild-type-like ascus formation, echoing previous results in the fungi *N. crassa* and *F. graminearum* (Alexander et al., 2008; Son et al., 2017). With respect to the Argonaute proteins, our results indicate that Sms2 is another key mediator of the early steps of meiocyte initiation, while Qde2 is (like Dcl2) dispensable for ascus formation and development.

The analysis of the double mutants does not delineate a simple epistatic/redundancy relationship between the four RNAi factors. The *dcl1 dcl2* double mutant displays a much stronger effect on meiocyte/ascus differentiation than either of the single mutants. Thus, Dcl2, plays also a role in this process but to a lesser extent than Dcl1. The phenotype of this double mutant shows also that the two dicer proteins operate within the same pathway with a minor contribution of Dcl2, like its *F. graminearum* homolog (Son et al., 2017; Zeng et al., 2018). It is the same for the two Argonaute proteins with a minor contribution of Qde2. Moreover, comparison of the *dcl1* and *sms2* single mutants indicates a different phenotype for *dcl1* than for *sms2*: at day 6, almost all *sms2* perithecia contain asci/meiocytes and spores, while 80% of the *dcl1* perithecia contain neither asci nor spores. Moreover, the perithecia of both mutants contain only 1 to 10 asci, versus over 100 in wild type. This suggests that Dcl1 (and to a lesser extent Dcl2) is primarily already involved in the transition from the vegetative to the sexual cycle. Once the vegetative/sexual transition has started, the dicer proteins (essentially Dcl1) and downstream the Argonaute proteins (mainly Sms2) are further required to produce wild-type ascus numbers. The two double mutants *dcl1 dcl2* and *sms2 qde2* are sterile but likely not at the same development stage. Our hypothesis is that *dcl1 dcl2* is not able to initiate the sexual cycle while *sms2 qde2* is able to initiate the sexual cycle but is unable to produce asci. Such independent roles of Dicer and Argonaute proteins have been reported in other systems, and could rely on the presence of other small, Dicer-independent, dsRNAs (Pong and Gullerova, 2018). The identified roles of Dicer and Argonaute proteins during meiocyte/ascus differentiation could be explained by a role of the RNAi pathway in the regulation of the gene expression needed for the transition from the vegetative program to the sexual cycle.

### RNAi Components Mediate Chromosome Axis Length During Meiotic Prophase

Meiotic chromosomes are highly organized structures. Each chromatid is organized into a linear array of loops, the bases of which comprise the axis, and sister chromatids are co-oriented with their axes tightly conjoined [reviewed in Zickler and Kleckner (2015)]. This organization emerges at the onset of meiotic prophase.

Analysis of the *dcl1*, *dcl2*, and *qde2* null mutants reveals the corresponding proteins as new players in the determination of chromosome-axis length. In all three cases, axis/SC lengths are increased when compared to wild type, indicating a change in the loop/axis relationship. Similar increased axis length has previously been reported in mutants lacking meiosis-specific cohesins SMC1Beta and Rec8, as well as other axis components like Spo76/Pds5, which all play key roles in the loop/axis relationship (e.g., Viera et al., 2020; Song et al., 2021). Several hypotheses could explain the observed changes in axis lengths. As a primary function of RNAi is to regulate gene expression, one hypothesis could be that Dicer and Argonaute proteins are involved in fine tuning of the expression of genes coding for proteins involved in meiotic chromosome axis morphogenesis. In *F. graminearum*, expression of *Rec8* is strongly reduced in the *Fgdlc1* mutant (Zeng et al., 2018) and lower Rec8 expression is accompanied by shorter chromosome axes in *S. cerevisiae* (Song et al., 2021) and with defects in SC organization in mouse (Murdoch et al., 2013).

Alternatively, RNAi proteins could regulate the chromatin state *per se*, by altering deposition of epigenetic marks for instance, as shown in *S. pombe* (Volpe et al., 2002), which could in turn affect chromatin compaction and/or the DNA loop sizes (e.g., in *Tetrahymena*; Wei et al., 1999). Consistent with this latter hypothesis is the observation that the *A. thaliana*, Dicer-like1 mutant (with other partner proteins) displays decondensed chromatin at the pachytene stage of meiotic prophase I (Oliver et al., 2017). Analogously, we find that *Sordaria* Dcl1, and especially Sms2, are required for wild-type like chromatin compaction. In wild type, DAPI staining indicates that chromatin is thin and smooth at zygotene and early pachytene, more compact at mid-pachytene and finally diffuse at late pachytene (De Muyt et al., 2014). In contrast, in the *dcl1* and *sms2* mutants, chromatin appears diffuse from zygotene throughout pachytene. Abnormal chromatin organization, globally or locally, could also explain the premature SC disorganization observed in these mutants. We cannot, however, exclude that, alternatively, RNAi components could play unconventional roles, e.g., to directly modulate either the axis status *per se* or the axis/loop organization, by interacting with axis proteins. Further studies are required to distinguish among these possible mechanisms.

### The RNAi Factors Are New Players in Crossover Patterning and Interference

One of the most fascinating aspects of meiosis is the highly regulated process that determines the number and positions of COs along homologs. Mutant phenotypes of the four analyzed RNAi factors provide new perspectives from which to consider the nature of these processes and the possible roles of RNAi. We find that when Dcl1, Dcl2, and Qde2 are absent, the number of crossovers is increased. This effect is attributable to the observed increased axis length, to which CO number is known to be proportional in *Sordaria* (Supplementary Figure 4A), and in a wide variety of other organisms (Wang et al., 2019). In addition, in the three corresponding mutants, the spatial patterning of COs along the homologs is altered when compared to wild type. There is, however, no loss of the “obligatory CO,” implying that CO designation *per se* remains robust.

We suspect that the altered CO distribution along homologs is related to the fact that in some chromosome regions SC formation is delayed, or hindered by entanglements into other chromosomes or homologs, channeling CO designation into regions where SC elongation progresses normally. We showed previously that CO patterns are determined by a designation and interference process that precedes SC formation, which is a concomitant downstream outcome (Zhang et al., 2014b). Aberrant synapsis and entanglements are likely to inhibit the implementation of these coordinate events in the mutants.

Alternatively, or in addition, the four RNAi proteins could regulate CO patterns directly, e.g., by alterations of the chromatin *per se* via epigenetic mark deposition. Roles for RNAi factors and epigenetic marks in recombination have been identified in several cases. In the fission yeast *S. pombe*, depletion of Dcr1, Ago1 or the histone methyl-transferase Clr4 leads to increased recombination in the pericentromeric region of chromosome III (Ellermeier et al., 2010). The same phenotype was observed in the *dcr1–5* point mutant in which the RNase III endonuclease domain was mutated specifically, arguing that Dcr1 role is directly correlated to its ability to dice dsRNAs (Ellermeier et al., 2010). However, in *S. pombe*, the increase in centromeric crossovers is accompanied by an increase in detectable DSBs, which is not the case in the *Sordaria dcl1* mutant where the number of Mer3 foci, which mark the sites of DSB interactions upon which CO patterning operates, is similar to wild type. Changes in epigenetic marks have also been linked to changes in recombination levels in *A. thaliana*: mutants with defective H3K9 and non-CG DNA methylation show increased crossover formation, while mutants with decreased CG DNA methylation show decreased recombination (Underwood et al., 2018). Finally, the Dicer and Argonaute proteins could, alternatively, control the expression of genes involved in the regulation of interference-sensitive crossovers.

### Why Do the *dcl1* and *sms2* Mutants Show so Many Abnormal Ascospores?

While *dcl2* and *qde2* mutants form regularly eight-spored asci, *dcl1*, and *sms2* mutants exhibit large numbers of abnormally shaped and aborted ascospores. Defective spore formation in *sms2* can be explained, at least in part, by the observed pairing, SC and CO defects, which, in turn, could lead to defective homolog segregation at anaphase I. However, the presence of so many abnormal spores in *dcl1* is an unexpected finding because the mutant exhibits normal pairing, SC formation, CO formation, including the obligatory CO that enables regular homolog segregation, and no chromosome breakage. Nevertheless, lagging chromosomes are visible in some anaphase I and II spindles (Supplementary Figure 5), which likely lead to defective chromosome segregation, which, in turn, would lead to the formation of abnormal spores. The involvement of the RNAi partners in chromosome segregation is a highly conserved feature. Dicer has been shown to be required for regular chromosome segregation during both mitosis and meiosis in fission yeast; the *dcr1* mutant exhibits lagging chromosomes during both divisions (Provost et al., 2002; Hall et al., 2003). Segregation defects during mitosis have been also uncovered in maize, *Drosophila*, *C. elegans* and mouse *dicer* and *ago* mutants (Deshpande et al., 2005; Harfe et al., 2005; Claycomb et al., 2009; Singh et al., 2011). Our results add a new organism to this list and show that *Sordaria* RNAi factors have, therefore additional post-meiotic functions. We also note that segregation defects during *Sordaria* mitotic divisions cannot be excluded because they might not occur in all nuclei and would thus not be detected in the multinuclear vegetative hyphal cells. Interestingly, the described defects in chromosome segregation are often correlated with defects in centromere composition, and/or mis-localization of centromeric marker proteins (Hall et al., 2003; Deshpande et al., 2005; Claycomb et al., 2009; Singh et al., 2011; Huang et al., 2015). These centromeric defects could be linked to a prominent role of RNAi in maintenance of centromeric and peri-centromeric heterochromatin state as described in *S. pombe* [reviewed in Goto and Nakayama (2012)].

Taken together, our results highlight the importance of the RNAi pathway in regulating key steps of the *Sordaria* sexual cycle. The importance of RNAi for sexual reproduction is widely conserved. However, our results reveal that both Dicer and Argonaute proteins are also specifically required during meiotic prophase, with direct or indirect roles in basic chromosome structure (through loop/axis modeling) and on the number plus localization of crossovers along the homologs. The molecular targets of those activities remain to be defined. We anticipate, however, that their elucidation will reveal important new aspects of the molecular activity of the analyzed RNAi factors.

## Data Availability Statement

The data presented in the study are deposited in the European Nucleotide Archive (ENA) repository, accession number PRJEB44726.

## Author Contributions

RD and EE initiated the project. CG, KB, SB, RD, and DZ conducted the experiments and collected data. CG, KB, SB, DZ, LZ, and EE performed analyses. CG and DZ wrote the manuscript. All authors contributed to the article and approved the submitted version.

## Conflict of Interest

The authors declare that the research was conducted in the absence of any commercial or financial relationships that could be construed as a potential conflict of interest.

## Footnotes

1 https://www.ncbi.nlm.nih.gov/Structure/cdd/wrpsb.cgi

2 http://www.sbg.bio.ic.ac.uk/phyre2

3 https://imagej.nih.gov/ij/

## Supplementary Material

Supplementary Figure 1 Dicer and Argonaute proteins: domains and expression. **(A)** Conserved domain positions across species in the two proteins. For Dicer, DEXDc, DEAD-like helicases superfamily; HeliCc, helicase superfamily c-terminal domain; Dimerization, Dicer dimerization domain; PAZ, named after the proteins Piwi Argonaute and Zwille; RNaseIII, Ribonuclease III family; dsRBD, double-stranded RNA binding motif. For Argonaute, PIWI, C-terminal portion which provides anchoring of the guide RNA and the catalytic site for slicing; Nter, N-terminal, which core fold closely resembles the catalytic domain of the replication-initiator protein Rep; Linker, Linker domain. **(B)** Time course expression of two meiotic genes (*SPO11* and *MER2*) compared with the *DICER* and *ARGONAUTE* genes during *Sordaria* vegetative et sexual cycles. At day 1, the mycelium has invaded the growing plate. At day 2 (graph) protoperithecia develop on the mycelium; day 3 and 4, perithecia contain mostly croziers and young asci (meiotic prophase); day 5 and 6, all perithecia contain asci at different steps of meiosis and post-meiotic mitosis; day 7 to 10, most asci contain mature ascospores. Fold changes and 95 % confidence interval (CI) are indicated. Fold changes are expressed relative to day 1. *SPO11* and *SMS2* at day 2, and all genes after day 2 have a fold change with a *p*-value < 0.05. Exact p-values and data necessary for the construction of the figure are shown in Supplementary Table 2.

Supplementary Figure 2 Vegetative growth and ascus formation. **(A)** Representative picture of mycelial growth 5 days after inoculation (right) and drawing of how measurements were made. **(B)** Growth curve of all mutants and double mutants analyzed in this study. Both growth rates and appearance of protophecia are synchronous in all mutants and are also identical to wild type. **(C)** Histograms of the percentage of perithecia with at least 1 ascus with spores in the simple *dcl1* and *dcl2* mutants and in all double mutants. The two other mutants *dcl2* and *qde2* were not included because they show the same percentage of asci with ascospores as wild type (see Supplementary Figure 3A). Numbers indicate the number of perithecia counted for each mutant.

Supplementary Figure 3 Sporulation phenotypes of four single mutants and three double mutants six days after inoculation. **(A)** *dcl2* and *qde2* show a majority of eight-spored asci with either black mature ascospores or white/grey ascospores that are not yet mature. Note that only half of the asci of each perithecium are presented. **(B,C)** Perithecia of *dcl1* **(B)** and *sms2* **(C)** contain only few asci and even fewer asci with eight mature ascospores. The other asci either contain a mixture of mature and abnormal ascospores or abort before sporulation. **(D)** Examples of perithecia of three double mutants: *dcl1 qde2* (left), *sms2 dcl2* (middle) which contain only few asci with even fewer spored-asci and *qde2 dcl2* (right) with eight-spored asci like wild type.

Supplementary Figure 4 CO and SC length correlation plus synapsis defects in mutants. **(A)** Linear regression analysis between the number of Hei10 foci per nucleus and the SC length in micron per nucleus in wild type, *dcl1*, *dcl2*, and *qde2*. For each nucleus the number of Hei10 foci is plotted against the total length of the SC. Both coefficient of determination (R^2^) and the regression line are indicated in the graph. **(B)** Mer3-GFP foci in wild-type (left) and *dcl1* (right) leptotene nuclei. **(C)** Zygotene nuclei of *qde2* (left) and *dcl2* (middle) with largely open non-synapsed regions (arrows). Note that half of the green homolog in the *qde2* nucleus remains completely unsynapsed (green arrow). In contrast, at the same stage, as the *dcl2* nucleus, the unsynapsed homologous segments of the WT zygotene are much closer (red arrows in both nuclei). **(D)** Example of asynchronous SC formation in *dcl2*. All homologs are either completely synapsed or with only a short unsynapsed segment (yellow homolog) when the red homolog exhibits a large open segment without any sign of entanglement. Bars, 2 μm.

Supplementary Figure 5 Chromosome segregation defects in *sms2* and *dcl1* mutants. **(A–C)** *sms2* mutants. **(A)** Seven bivalents at diplotene. **(B)** Three lagging chromosomes are visible in this anaphase I spindle (red arrows). **(C)** Lagging chromosomes are also visible during anaphase of the second meiotic division (arrow). **(D,E)** *dcl1* mutant. **(D)** Irregular chromosome segregation during anaphase of the second meiotic division. In the left spindle, 4 or 5 chromosomes are at one pole and 9 at the other pole, when a 7/7 segregation is expected. Segregation is also defective in the right spindle (arrow). **(E)** Irregular segregation leads to pear-shaped telophase nuclei (arrow). Bars, 2 μm.
