# Supplementary figures and images for: RNAi-Related Dicer and Argonaute Proteins Play Critical Roles for Meiocyte Formation, Chromosome-Axes Lengths and Crossover Patterning in the Fungus Sordaria macrospora
Source: Front Cell Dev Biol. 2021 Jun 28;9:684108. doi: 10.3389/fcell.2021.684108 (PMC8274715; doi:10.3389/fcell.2021.684108)

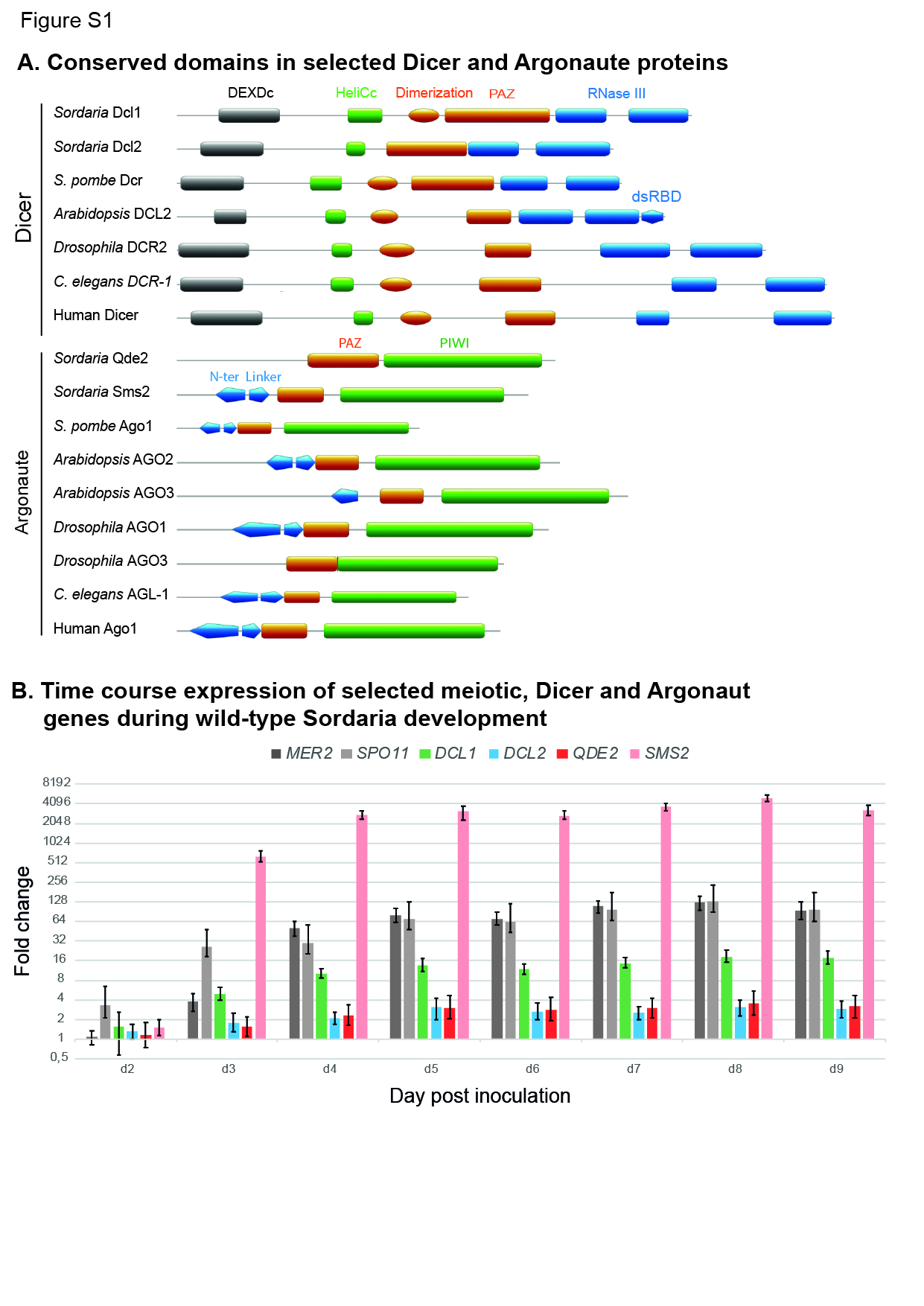

Supplement: Supplementary Figure 1 — Dicer and Argonaute proteins: domains and expression. (A) Conserved domain positions across species in the two proteins. For Dicer, DEXDc, DEAD-like helicases superfamily; HeliCc, helicase superfamily c-terminal domain; Dimerization, Dicer dimerization domain; PAZ, named after the proteins Piwi Argonaute and Zwille; RNaseIII, Ribonuclease III family; dsRBD, double-stranded RNA binding motif. For Argonaute, PIWI, C-terminal portion which provides anchoring of the guide RNA and the catalytic site for slicing; Nter, N-terminal, which core fold closely resembles the catalytic domain of the replication-initiator protein Rep; Linker, Linker domain. (B) Time course expression of two meiotic genes (SPO11 and MER2) compared with the DICER and ARGONAUTE genes during Sordaria vegetative et sexual cycles. At day 1, the mycelium has invaded the growing plate. At day 2 (graph) protoperithecia develop on the mycelium; day 3 and 4, perithecia contain mostly croziers and young asci (meiotic prophase); day 5 and 6, all perithecia contain asci at different steps of meiosis and post-meiotic mitosis; day 7 to 10, most asci contain mature ascospores. Fold changes and 95 % confidence interval (CI) are indicated. Fold changes are expressed relative to day 1. SPO11 and SMS2 at day 2, and all genes after day 2 have a fold change with a p-value < 0.05. Exact p-values and data necessary for the construction of the figure are shown in Supplementary Table 2. [file Image_1.TIF]

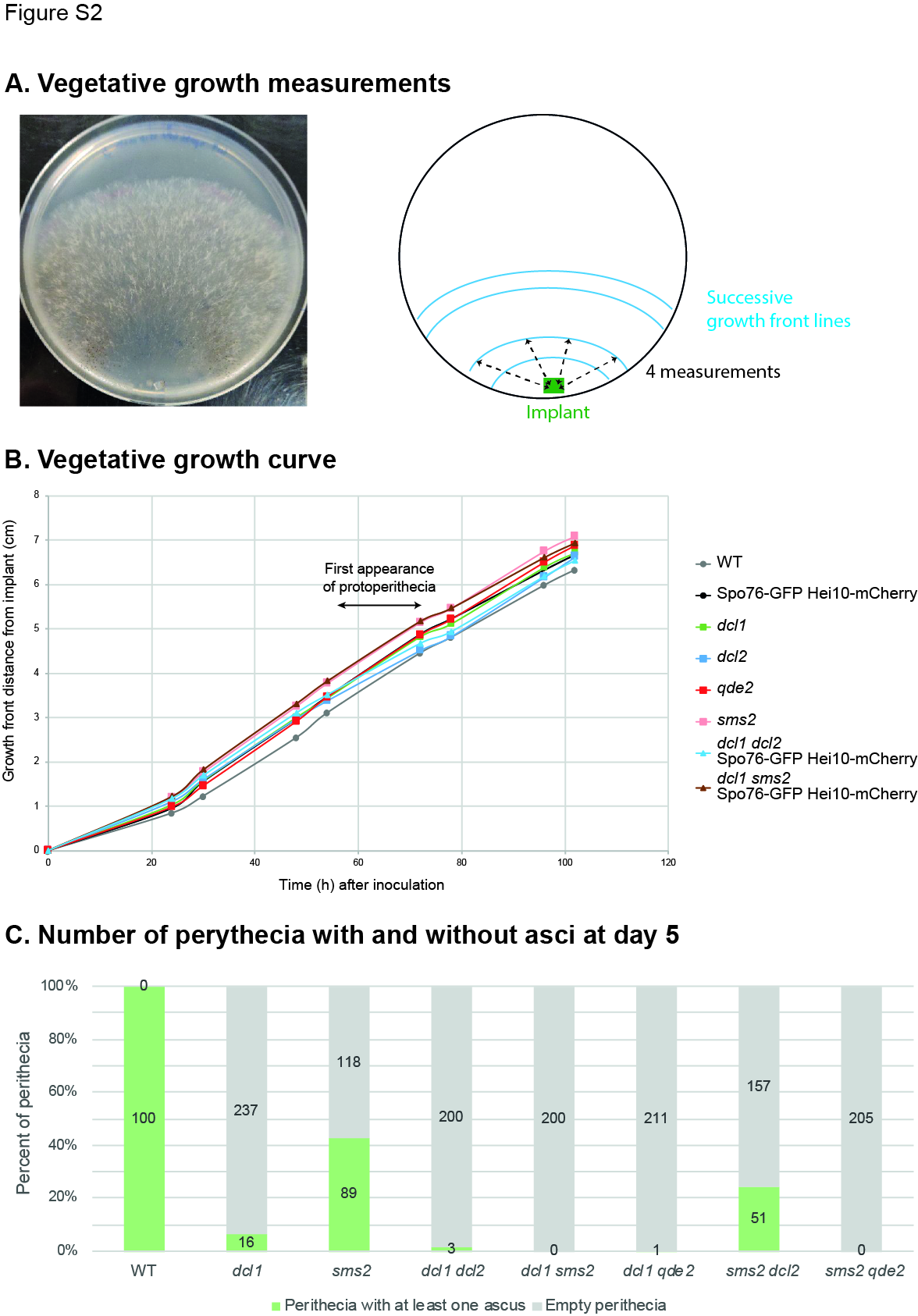

Supplement: Supplementary Figure 2 — Vegetative growth and ascus formation. (A) Representative picture of mycelial growth 5 days after inoculation (right) and drawing of how measurements were made. (B) Growth curve of all mutants and double mutants analyzed in this study. Both growth rates and appearance of protophecia are synchronous in all mutants and are also identical to wild type. (C) Histograms of the percentage of perithecia with at least 1 ascus with spores in the simple dcl1 and dcl2 mutants and in all double mutants. The two other mutants dcl2 and qde2 were not included because they show the same percentage of asci with ascospores as wild type (see Supplementary Figure 3A). Numbers indicate the number of perithecia counted for each mutant. [file Image_2.TIF]

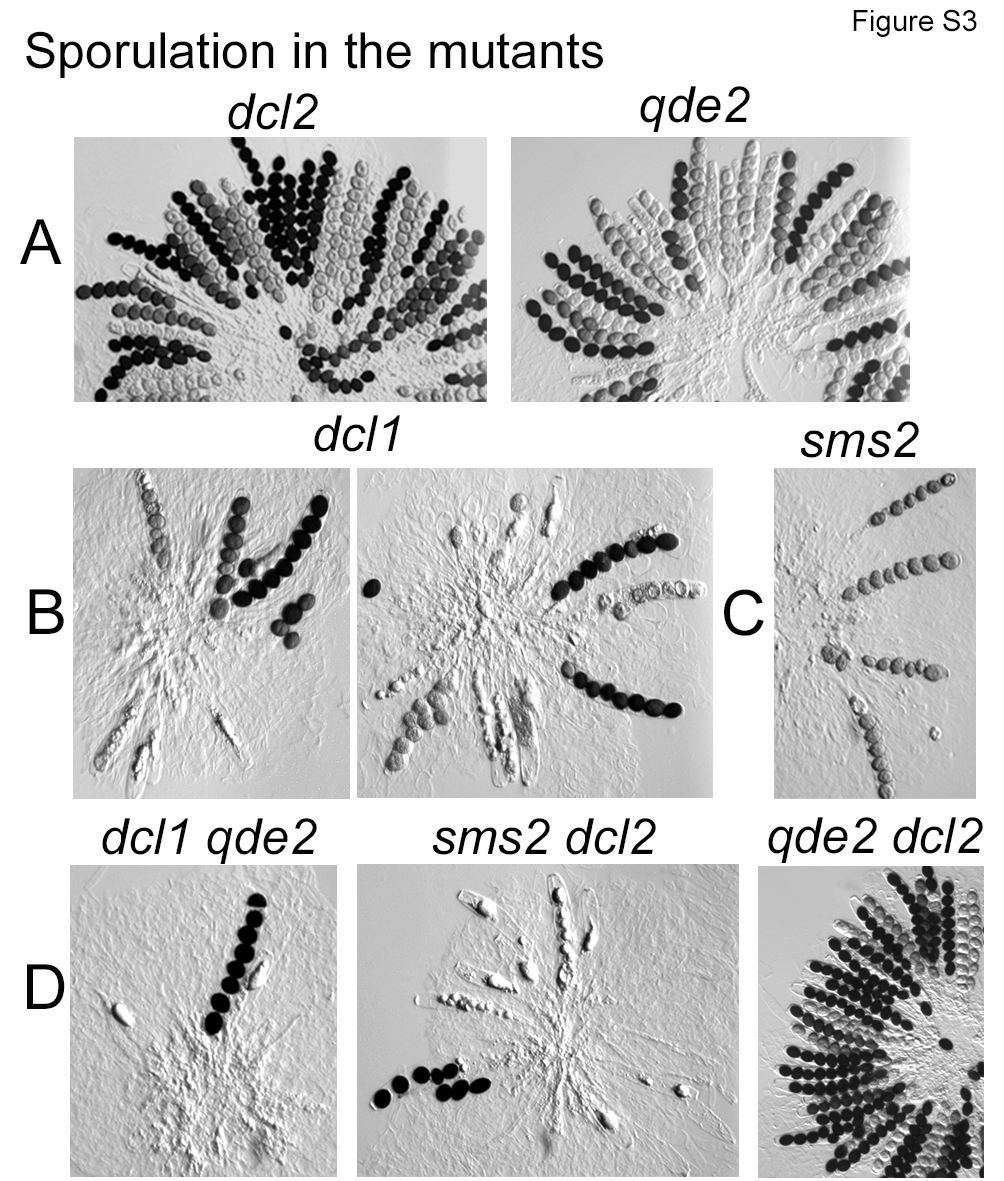

Supplement: Supplementary Figure 3 — Sporulation phenotypes of four single mutants and three double mutants six days after inoculation. (A) dcl2 and qde2 show a majority of eight-spored asci with either black mature ascospores or white/grey ascospores that are not yet mature. Note that only half of the asci of each perithecium are presented. (B,C) Perithecia of dcl1 (B) and sms2 (C) contain only few asci and even fewer asci with eight mature ascospores. The other asci either contain a mixture of mature and abnormal ascospores or abort before sporulation. (D) Examples of perithecia of three double mutants: dcl1 qde2 (left), sms2 dcl2 (middle) which contain only few asci with even fewer spored-asci and qde2 dcl2 (right) with eight-spored asci like wild type. [file Image_3.TIF]

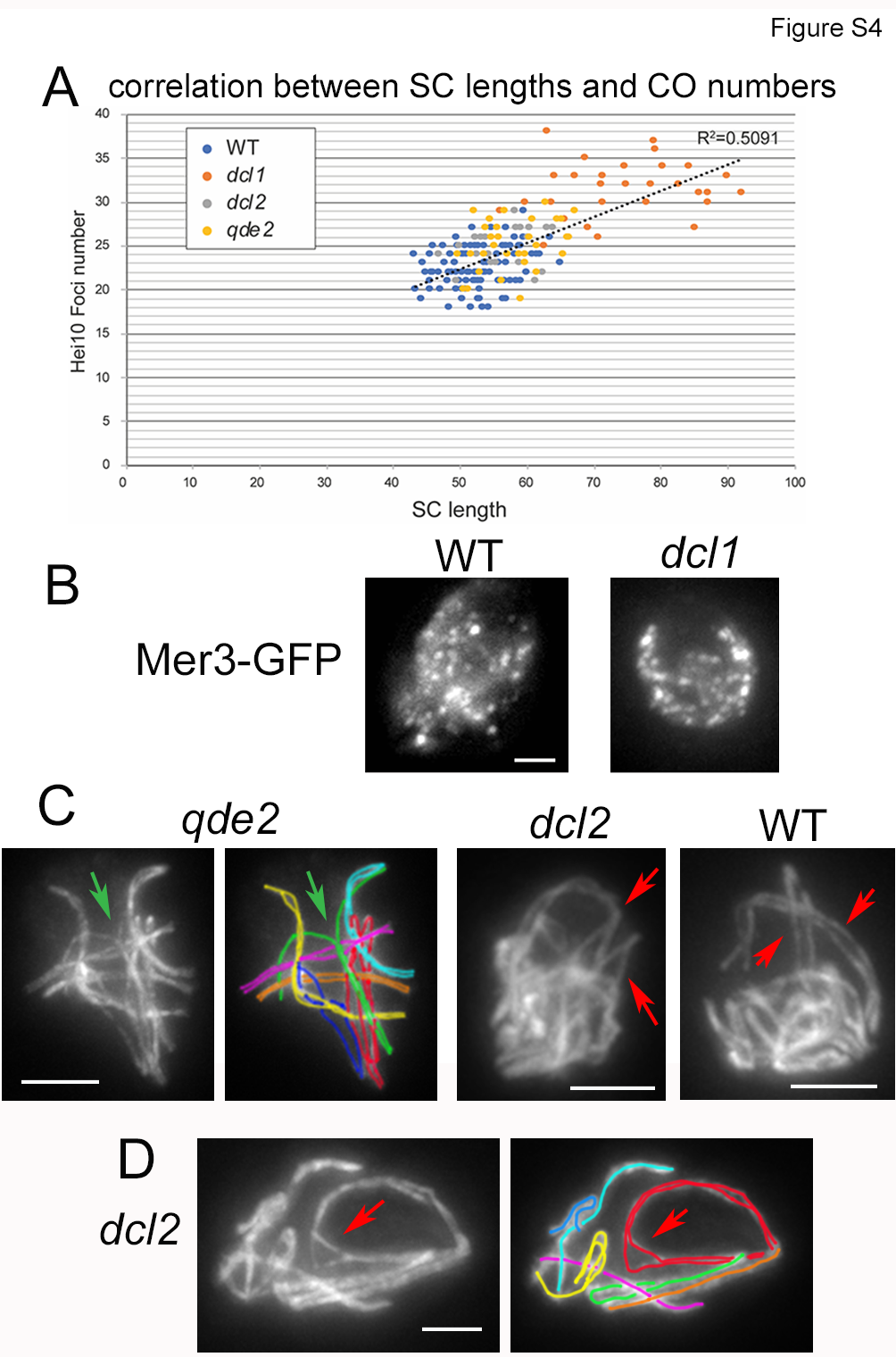

Supplement: Supplementary Figure 4 — CO and SC length correlation plus synapsis defects in mutants. (A) Linear regression analysis between the number of Hei10 foci per nucleus and the SC length in micron per nucleus in wild type, dcl1, dcl2, and qde2. For each nucleus the number of Hei10 foci is plotted against the total length of the SC. Both coefficient of determination (R2) and the regression line are indicated in the graph. (B) Mer3-GFP foci in wild-type (left) and dcl1 (right) leptotene nuclei. (C) Zygotene nuclei of qde2 (left) and dcl2 (middle) with largely open non-synapsed regions (arrows). Note that half of the green homolog in the qde2 nucleus remains completely unsynapsed (green arrow). In contrast, at the same stage, as the dcl2 nucleus, the unsynapsed homologous segments of the WT zygotene are much closer (red arrows in both nuclei). (D) Example of asynchronous SC formation in dcl2. All homologs are either completely synapsed or with only a short unsynapsed segment (yellow homolog) when the red homolog exhibits a large open segment without any sign of entanglement. Bars, 2 μm. [file Image_4.TIF]

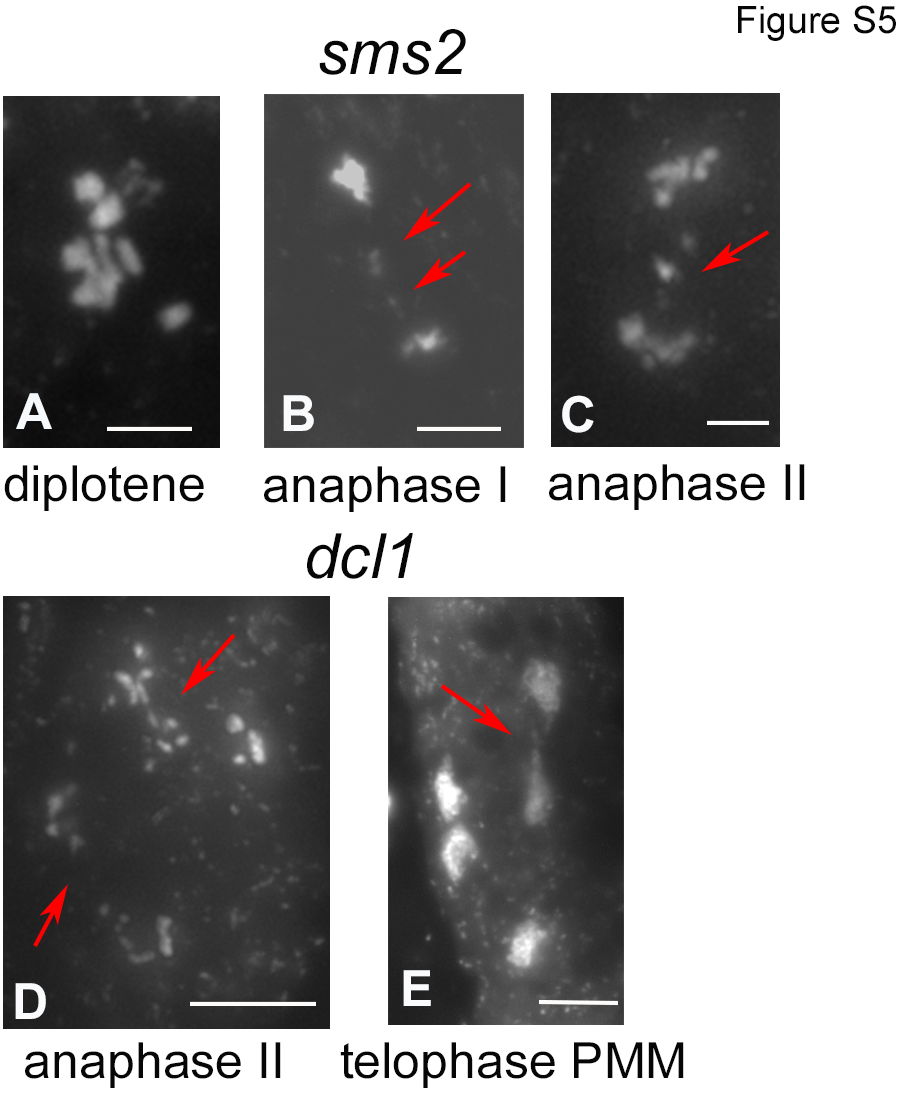

Supplement: Supplementary Figure 5 — Chromosome segregation defects in sms2 and dcl1 mutants. (A–C) sms2 mutants. (A) Seven bivalents at diplotene. (B) Three lagging chromosomes are visible in this anaphase I spindle (red arrows). (C) Lagging chromosomes are also visible during anaphase of the second meiotic division (arrow). (D,E) dcl1 mutant. (D) Irregular chromosome segregation during anaphase of the second meiotic division. In the left spindle, 4 or 5 chromosomes are at one pole and 9 at the other pole, when a 7/7 segregation is expected. Segregation is also defective in the right spindle (arrow). (E) Irregular segregation leads to pear-shaped telophase nuclei (arrow). Bars, 2 μm. [file Image_5.TIF]
