# Supplementary material for: RNAi-Related Dicer and Argonaute Proteins Play Critical Roles for Meiocyte Formation, Chromosome-Axes Lengths and Crossover Patterning in the Fungus Sordaria macrospora
Source: Front Cell Dev Biol. 2021 Jun 28;9:684108. doi: 10.3389/fcell.2021.684108 (PMC8274715; doi:10.3389/fcell.2021.684108)
Supplement: Supplementary file 6 [file Table_1.DOCX]

Table S1 : Primers used for RT-qPCR experiments

| gene | Gene nomenclature | Primer name | Sequence^a^ |
| --- | --- | --- | --- |
| *AS1* | SMAC_00248 | AS1f-SM | ggctgacgaatac/aacgccg |
|  |  | AS1r-SM | ccaaggtcgaggaga/gcatc |
| *CIT1* | SMAC_02401 | CIT1f-SM | ctactcttccaagactcag/actc |
|  |  | CIT1r-SM | cgatcttggaaccgtgctc/cttg |
| *GPD* | SMAC_06203 | GPDE1E2 | cattgagcccaagtacgct/ga |
|  |  | GPDE2reverse | gtcgcgctcagtgtagaacttga |
| *H2A* | SMAC_08324 | H2Af-SM | ccaagaacgcgcaatc/ccgc |
|  |  | H2Ar-SM | gcccgcaagctcgagaatttcg |
| *PDF2* | SMAC_01919 | PDF2f_Sm | cacttgctttgctatcacg/acc |
|  |  | PDF2r_Sm | gaaggcgacgaagaacacc |
| *TBP* | SMAC_03405 | TBPf-SM | catgacccctactctacag/aacatc |
|  |  | TBPr-SM | gcaaaacgcttggggttata/ctcg |
| *TIP42* | SMAC_08092 | TIPf_Sm | gtttgagaatgtgaagacg/aaac |
|  |  | TIPr_Sm | cttgataagcggcagcaga |
| *UBC* | SMAC_07925 | UBCf_Sm | tccatcaacggtccattcg |
|  |  | UBCr_Sm | gatggtcttgccagtca/aagt |
| *MER2* | SMAC_02785 | E1ASY2 | gcctcttatcgaagcag/cgc |
|  |  | E2ASY2 | cgcttcgggttcgctcttt |
| *SPO11* | SMAC_03194 | SPO11f3 | cgtcattgagaaggag/gcc |
|  |  | SPO11r3 | cgggatatccttttcc/agtc |
| *DCL1* | SMAC_00946 | 946f1 | cgttgaggatttcacct/atac |
|  |  | 946r1 | aattgtcacccattccagtc |
| *DCL2* | SMAC_06757 | 6757f1 | cagcttcggcattat/ttcc |
|  |  | 6757r1 | acaaacttaggccgccact |
| *QDE2* | SMAC_03832 | 3832f2 | cagttcctggtggtggttcta |
|  |  | 3832r2 | ttgatgttgtggtttgtgcc |
| *SMS2* | SMAC_08605 | 8605f1 | aaggataaggcgggatgtga |
|  |  | 8605r1 | gaatgtggtgacgcttggtg |

^a^ : slash in primer sequence indicates intron position.
