# Supplementary material for: RNAi-Related Dicer and Argonaute Proteins Play Critical Roles for Meiocyte Formation, Chromosome-Axes Lengths and Crossover Patterning in the Fungus Sordaria macrospora
Source: Front Cell Dev Biol. 2021 Jun 28;9:684108. doi: 10.3389/fcell.2021.684108 (PMC8274715; doi:10.3389/fcell.2021.684108)
Supplement: Supplementary file 7 [file Table_2.docx]

Table S2 : primers used for the construction of the deletion cassettes

| Primer name | Primer sequence^a^ | Primer use |
| --- | --- | --- |
| 946_5f | gtaacgccagggttttcccagtcacgacggcgcgccgctggagaggcaggtcc | Amplification of the 5’ flanking sequence of *DCL1* |
| 946_5r | cagcagccgcacgcggcgcatctcggaacagtgacggcctgttgttcttttcc | Amplification of the 5’ flanking sequence of *DCL1* |
| 946_3f | tccttctttctagctagaggatcctctacgccagttcattacatatttcggcac | Amplification of the 3’ flanking sequence of *DCL1* |
| 946_3r | gcggataacaatttcacacaggaaacagcggcgcgcctggatcacatgggttatggg | Amplification of the 3’ flanking sequence of *DCL1* |
| 6757_5f | gtaacgccagggttttcccagtcacgacggcgcgccgcaaatgggttgagtggtgg | Amplification of the 5’ flanking sequence of *DCL2* |
| 6757_5r | cagcagccgcacgcggcgcatctcggaacggcggctgatgaatcgacggttttg | Amplification of the 5’ flanking sequence of *DCL2* |
| 6757_3f | tccttctttctagctagaggatcctctacaagagtcaggagacggtccctatc | Amplification of the 3’ flanking sequence of *DCL2* |
| 6757_3r | gcggataacaatttcacacaggaaacagcggcgcgccgcttcccaccccgttcccttc | Amplification of the 3’ flanking sequence of *DCL2* |
| 3832_5f | gtaacgccagggttttcccagtcacgacggcgcgcccgccctcagtccaac | Amplification of the 5’ flanking sequence of *QDE2* |
| 3832_5r | cagcagccgcacgcggcgcatctcggaacttgtggtactagtggtgacag | Amplification of the 5’ flanking sequence of *QDE2* |
| 3832_3f | tccttctttctagctagaggatcctctactgggcatggagatggaattgaag | Amplification of the 3’ flanking sequence of *QDE2* |
| 3832_3r | gcggataacaatttcacacaggaaacagcggcgcgccaggcaggacctttacgg | Amplification of the 3’ flanking sequence of *QDE* |
| 8605_5f | gtaacgccagggttttcccagtcacgacggcgcgccttggagccattattttgg | Amplification of the 5’ flanking sequence of *SMS2*  Incomplete deletion |
| 8605_5r | cagcagccgcacgcggcgcatctcggaactggcaggatcgtgtccgagag | Amplification of the 5’ flanking sequence of *SMS2*  Incomplete deletion |
| 8605_3f | tccttctttctagctagaggatcctctactcttgggccgctttgacg | Amplification of the 3’ flanking sequence of *SMS2* |
| 8605_3r | gcggataacaatttcacacaggaaacagcggcgcgccgcgatatctcacctctgagttc | Amplification of the 3’ flanking sequence of *SMS2* |
| RNA1_5f | gtaacgccagggttttcccagtcacgacggcgcgccttggtcaaggataggaagtag | Amplification of the 5’ flanking sequence of *SMS2*  complete deletion |
| RNA1_5r | cagcagccgcacgcggcgcatctcggaacggagaaggatatgagatggcc | Amplification of the 5’ flanking sequence of *SMS2*  complete deletion |

^a^ : nucleotides corresponding to pRS426 are in red, AscI site is in green, nucleotides correponding to the gene of interest are in black, nucleotides corresponding to the hgromycine resistance cassette are in blue.
